# EUS-guided interventional therapies for pancreatic diseases

**DOI:** 10.3389/fmed.2023.1329676

**Published:** 2024-01-08

**Authors:** Rongmin Xu, Kai Zhang, Nan Ge, Siyu Sun

**Affiliations:** Department of Gastroenterology, Shengjing Hospital of China Medical University, Shenyang, Liaoning, China

**Keywords:** EUS, pancreatic diseases, treatment, drainage, ablation, celiac plexus neurolysis

## Abstract

Endoscopic ultrasound (EUS) is an integrated diagnostic technique merging endoscope and ultrasound to examine the digestive system. EUS has emerged as a primary diagnostic method for pancreatic diseases due to its distinctive benefits. Over the past four decades, EUS has undergone a transformation, shifting its role from primarily diagnostic to increasingly therapeutic. Additionally, in recent years, EUS has emerged as an increasingly prominent adjunctive or alternative approach to conventional surgical interventions. This review provides a comprehensive analysis of current technological approaches in the treatment of pancreatic diseases. The dynamic interplay with diverse therapeutic approaches has reinvigorated EUS and shaped its trajectory in the management of pancreatic diseases.

## 1 Introduction

Endoscopic ultrasound (EUS) (1) is an integrated diagnostic technique merging endoscope and ultrasound to examine the digestive system. It involves a miniature high-frequency ultrasound probe at the distal end of the endoscope. The endoscope enables direct visualization of mucosal lesions in the digestive system. Moreover, the ultrasound facilitates real-time scanning to generate images of the gastrointestinal structures and surrounding extramural structures, thereby augmenting the diagnostic capabilities. EUS presents distinctive advantages when compared to other techniques: it provides close proximity to the tissue, reducing interference from subcutaneous tissue, bones and gas. The utilization of high-resolution, real-time imaging facilitates the identification of minute lesions (2). As a consequence of these unparalleled advantages, EUS has emerged as a primary diagnostic approach for pancreatic lesions. Over the past four decades, EUS has undergone a transformation, shifting its role from primarily diagnostic to increasingly therapeutic. Additionally, in recent years, EUS has emerged as an increasingly prominent adjunctive or alternative approach to conventional surgical interventions. Currently, the therapy for pancreatic diseases comprises: Endoscopic ultrasound-guided drainage of pancreatic fluid collections, Endoscopic ultrasound-guided fine-needle injection (EUS-FNI), Endoscopic ultrasound-guided ablation, Endoscopic ultrasound-guided fiducial implantation and Endoscopic ultrasound-guided brachytherapy, Endoscopic ultrasound-guided celiac plexus block and neurolysis (EUS-CPB/CPN). This review provides a comprehensive interpretation of the current technological approaches, status, and developments in the field.

## 2 Endoscopic ultrasound-guided drainage of pancreatic fluid collections

Acute or chronic pancreatitis may give rise to the development of fluid collections within the pancreas and/or the peripancreatic area. Pancreatic fluid collections (PFCs) that persist for >4 weeks encompass pseudocysts, characterized by fluid accumulation, and walled-off necrosis (WON), defined by the presence of solid necrotic debris (3). When symptoms such as abdominal pain, nausea, vomiting, and infection occur, intervention is essential. Management modalities comprise EUS-guided drainage, esophagogastroduodenoscopy (EGD) -guided drainage, percutaneous drainage, and surgical intervention (4). EUS-guided drainage has been found to demonstrate comparable or potentially lower mortality rates and adverse events (AEs) compared to alternative approaches, making it the front-line treatment for pseudocysts and WON (5–11).

The stents used for drainage comprise double pigtail plastic stents (DPPS), self-expanding metal stent (SEMS) and lumen-apposing metal stent (LAMS). LAMS, featuring a dumbbell-shaped structure, offers ease of use compared to SEMS, leading to decreased procedure time and lower stent dislodgment (12). Moreover, LAMS provides a larger diameter that facilitates direct endoscopic necrosectomy (13). Considering the aforementioned advantages, LAMS has emerged as the preferred stent choice for endoscopic drainage among a significant number of gastroenterologists (14–16). Although possessing notable advantages, LAMS exhibits certain limitations. In comparison to DPPS, LAMS is linked to an increased susceptibility to bleeding, especially in the form of delayed bleeding manifesting 3–5 weeks post-intervention (17–20). Incorporating color Doppler ultrasound guidance during procedures has the potential to reduce the risk of intraoperative bleeding, however, it does not have any impact on the occurrence of delayed bleeding (21). Two potential explanations exist for the elevated bleeding rate observed in association with LAMS. One explanation postulates that while the lesion diminishes in size during drainage, the LAMS remains in place, causing friction between the stent and adjacent blood vessels, leading to the development of pseudoaneurysms and subsequent hemorrhage (22). Another explanation posits that the broader lumen diameter of LAMS facilitates the entry of low pH gastric acid into the cystic cavity, consequently stimulating the exposed intraluminal blood vessels and heightening the susceptibility to bleeding (23). Consequently, the European Society of Gastrointestinal Endoscopy (ESGE) guideline advocate removing stent within 4 weeks to reduce the risk of complications (24). Nonetheless, several studies have demonstrated that the incidence of AEs associated with LAMS is either unchanged or potentially reduced (25–27). Additionally, Amato et al. (28) demonstrated that a longer removal time did not increase the risk of AEs. Recent randomized controlled trials (RCTs) have not shown the superiority of LAMS over DPPS or an increased bleeding risk for WON (29–31), nor have they demonstrated superiority of LAMS over SEMS for PFCs (32).

The current literature provides evidence that the placement of LAMS with coaxial DPPS for the drainage of pseudocysts results in a noticeably elevated clinical success rate (33), reduced bleeding (34), infection rate (0% vs. 17%) (35), stent occlusion compared to LAMS without DPPS (36). Nevertheless, Shamah et al. (37) exhibit divergent perspectives on the results. They consider that LAMS with or without DPPS does not impact the clinical outcomes, AEs, and reintervention rates associated with PFCs drainage. With respect to the time of drainage, American Society for Gastrointestinal Endoscopy (ASGE) recommends avoiding drainage during the acute phase (< 4 weeks) and advocating for the procedure after the maturation of the cyst wall (≥ 4 weeks) to enhance safety (38). However, a recent meta-analysis revealed that there is no statistical difference in technical and clinical success rates between early drainage (< 4 weeks) and late drainage (39). More RCTs are needed to further compare the safety and efficacy of different stents as well as the time of drainage.

## 3 Endoscopic ultrasound-guided fine-needle injection (EUS-FNI)

EUS-FNI is a critical component of targeted tumor therapy, providing several benefits including the reduction of systemic AEs and enhancement of intratumoral drug concentrations. It can be employed for the treatment of pancreatic solid tumors and cystic lesions, encompassing specific applications such as chemotherapy, immunotherapy, and gene therapy (40) (Table 1).

**Table 1:** Characteristics of recently available studies evaluating EUS-FNI in patients with pancreatic solid tumors.

Author, year (reference)	No. of patients (stage)	Study type	Intratumoral injection agents (additional therapy)	Response	Median OS (months)
Chemotherapy					
Levy et al., 2017 (41)	36 (3 stage II, 20 stage III, 13stage IV)	Prospective	Gemcitabine	PR 25%, stable disease 57%	10.4
Immunotherapy					
Hirooka et al., 2017 (42)	15 (locally advanced)	Prospective	Zoledronate-pulsed dendritic cells (gemcitabine and αβT cells)	Stable disease 46.7%	11.5
Gene therapy					
Herman et al., 2013 (43)	304 (locally advanced)	Prospective	TNFerade Biologic (fluorouracil, external-beam radiotherapy, followed by maintenance gemcitabine ± erlotinib)	No difference	10.0
Nishimura et al., 2018 (44)	6 (NA)	Prospective	STNM01, a RNA oligonucleotide	NA	5.8
Fujisawa et al., 2022 (45)	22 (locally advanced)	Prospective	STNM01 (S-1 s-line therapy)	PR 4.5%, stable disease 68.2%	7.8

NA, not available; OS, overall survival; PR, partial response.

Chemotherapy involves the application of chemotherapeutic agents such as gemcitabine as well as oncolytic viruses (46). Gemcitabine has been utilized in the treatment of pancreatic cancer for more than two decades. EUS-FNI of gemcitabine has demonstrated favorable feasibility and safety in pancreatic cancer (41). However, its effectiveness in pancreatic cancer is constrained by its rapid clearance. To surmount this impediment, drug nanocarriers have been devised, incorporating active targeting to recognize tumor cells and passive targeting to improve drug penetration and retention at the tumor. This synergistic approach yields heightened anticancer effects and mitigates the emergence of drug resistance resulting from repeated administration (47). Various drug delivery systems, such as hydrogels, microspheres, nanoparticles, micelles, and drug eluting devices, have received considerable attention (48–52). Recently, a nanomedicine delivery system comprising gemcitabine and thermosensitive hydrogels has been developed. The therapeutic efficacy of this system was assessed in a murine model of pancreatic cancer through EUS-FNI. The results indicate that the system enables sustained drug release for 7 days, leading to a significant reduction in pancreatic cancer cell proliferation, invasion, and migration. It promotes cell apoptosis, resulting in a remarkable tumor weight reduction of up to 75.96%. Furthermore, it extends the overall survival (OS)of mice by 14.4 days, with minor AEs and no systemic toxicity based on pathology (53).

Immunotherapy is a therapeutic approach aimed at stimulating immune cells to induce tumor-specific immune responses, thereby indirectly inhibiting and eliminating tumor cells. Immune stimulation of lymphocytes and dendritic cells can be accomplished through EUS-FNI (54, 55). The results of a study assessing the therapeutic efficacy of comprehensive immunotherapy combined with intratumoral injection of zoledronate-pulsed dendritic cells, intravenous adoptive activated T lymphocyte and gemcitabine in unresectable locally advanced pancreatic carcinoma. Fifteen patients are involved, 7 experienced disease stabilization, with a majority demonstrating sustained clinical responses. Notably, patients with disease stability exhibited a significant increase in the CD8+/Treg ratio. The median survival time (MST) and progression-free survival (PFS) for the 15 patients were 12.0 and 5.5 months, respectively. Moreover, this study not only affirms the safety and feasibility of EUS-FNI but also elucidates its potential in inducing immune responses against pancreatic cancer (42).

EUS-FNI gene therapy includes both DNA and RNA (46). STNM01, an artificially synthesized double-stranded RNA oligonucleotide, exhibits selective inhibition of carbohydrate sulfotransferase-15 (CHST-15) expression. Previous studies have implicated an association between CHST-15 and unfavorable prognosis in pancreatic cancer (56). Nishimura et al. (44) conducted a study where they administered 250 nM of STNM01 to a cohort of 6 patients diagnosed with pancreatic cancer. The study observed reductions in tumor size and demonstrated no AEs, leading to an extension in OS. Nonetheless, the MST was observed to be a mere 5.8 months, and a decline in CHST-15 levels was observed in 50% of the patients. In a recent phase I/II clinical trial, a cohort comprising 22 patients diagnosed with pancreatic cancer was prospectively enrolled. Following a cyclical pattern with a biweekly dosing interval, the patients received STNM01 at varying concentrations of 250, 1,000, 2,500, or 10,000 nM. Encouragingly, no AEs were reported, and the study demonstrated a MST of 7.8 months. These significant findings substantiate the safety and feasibility of administration of STNM01 through EUS-FNI as an effective therapeutic modality for pancreatic cancer (45). All approaches provided evidence supporting the safety and efficacy of EUS-FNI as a treatment for pancreatic cancer. Significantly, there have been no RCTs that have shown any advantages of EUS-FNI compared to conventional therapy in the treatment of pancreatic cancer and the final results of a single large-scale RCT comparing the standard of care plus TNFerade Biologic versus standard of care alone, found that no difference between two groups (43). Future prospective research is needed to further compare the differences between various methods.

## 4 Endoscopic ultrasound-guided ablation

The field of EUS has made significant progress in the direction of intraluminal invasive therapeutic procedures. Notably, EUS-guided ablation has become an essential component in its developmental trajectory. EUS-guided ablation encompasses a wide array of procedures, such as EUS-guided ethanol ablation (EUS-EA), EUS-guided radiofrequency ablation (EUS-RFA), EUS-guided cryothermal ablation (EUS-CTA), EUS-guided photodynamic therapy (EUS-PDT), and EUS-guided laser ablation (EUS-LA). Collectively, these procedures offer a diverse range of therapeutic modalities guided by EUS (57) (Table 2).

**Table 2:** Characteristics of available studies evaluating EUS-guided ablation in patients.

Author, year (reference)	No. of patients	Study type	Disease type	Ablation methods	Outcome	Related adverse events rate%
Gan et al., 2005 (58)	25	Prospective	PCL	Ethanol	CR 35% (12 months)	0
Arcidiacono et al., 2012 (59)	22	Prospective	LAPC	CTA	Technical success rate 72.8%	18.2
Choi et al., 2015 (60)	4	Prospective	Pancreaticobiliary malignancies	PDT	Technical success rate 100%	0
Linghu et al., 2017 (61)	29	Prospective	PCN	Lauromacrogol	CR 37.9%, PR 31.0% (9 months)	10.3
Choi et al., 2018 (62)	33	Prospective	NET < 2 cm	Ethanol-lipiodol	CR 60% (42 months)	3.2
Matteo et al., 2018 (63)	9	Prospective	PC	LA	Technical success rate 100%	0
DeWitt et al., 2019 (64)	12	Prospective	LAPC	PDT	Technical success rate 100%	0
Bang et al., 2019 (65)	12	Prospective	LAPC	RFA	PAN26 (49.0), C30 (51.9), VAS (30.1), opioid 105.4 mg	41.7
Matsumoto et al., 2020 (66)	5	Prospective	NET < 2 cm	Ethanol	CR 80% (12 months)	0
Du et al., 2022 (67)	70	Prospective	PCN	Lauromacrogol	CR 51.4%, PR 25.7% (12 months)	3.6
So et al., 2023 (68)	97	Retrospective PSM	NET < 2 cm	Ethanol	CR 65%, PR 21.6%	3.4
Song et al., 2023 (69)	169	Retrospective PSM	PCL	Ethanol	CR 74%	13

PCL, pancreatic cystic lesion; LAPC, locally advanced pancreatic cancer; PC, pancreatic cancer; PCN, pancreatic cystic neoplasm; NET, neuroendocrine tumor; CTA, cryothermal ablation; PDT, photodynamic therapy; LA, laser ablation; RFA, radiofrequency ablation; CR, complete response; PR, partial response; PAN26, C30, European Organization for Research and Treatment of Cancer-Quality of life Questionnaire; VAS, Visual Analog Scale, a pain rating scale.

The early use of ethanol ablation by GAN et al. (58) and Jürgensen et al. (70) demonstrated the safety and feasibility of treating pancreatic cystic lesions (PCLs) and neuroendocrine tumors (NETs). A propensity score-matching (PSM) study was conducted to investigate the impact of EUS-EA on PCLs. The study included patients with enlarging suspected branch-duct intraductal papillary mucinous neoplasms (BD-IPMNs) or PCLs >3 cm. The findings revealed that EUS-EA was associated with decreased 10-year cumulative incidence rates of BD-IPMNs and a declining trend in surgical resection (SR), whereas its 10-year OS and disease-specific survival (DSS) were comparable to controls (69). In addition, paclitaxel, lipiodol, lauromacrogol, and gemcitabine, either alone or in combination, have been employed for the ablation of PCLs (61, 62, 67, 71). A meta-analysis included 15 studies comprising 524 patients and aimed to evaluate the efficacy of ethanol as a standalone agent compared to its combination with paclitaxel in ablating PCLs. The results demonstrated similar efficiency between the two approaches, yet the latter approach was associated with a higher occurrence of AEs (72). But a recent meta-analysis demonstrated opposite result (73). Due to the unpredictable malignant potential, the management of non-functional NETs measuring <2 cm has stirred controversy. So et al. (68) conducted a PSM study to evaluate the safety and efficacy of EUS-EA and surgery for non-functional NETs measuring <2 cm. The study findings revealed that EUS-EA exhibited a reduced incidence of complications, a shorter duration of hospitalization, and comparable OS and DSS. The results suggest that EUS-EA may serve as a preferred alternative for patients with non-functional small pancreatic NETs (66). It can also be used for pancreatic cancer ablation (74), but it is limited in acquiring pathology for further elucidation of the disease type.

EUS-RFA is a thermal therapy that employs a high-frequency electrical probe, with the purpose of generating and delivering heat to induce coagulative necrosis within the targeted tissue, ultimately resulting in the ablation of the targeted lesion (65). Existing literature suggests that RFA has the potential to generate cellular fragments, facilitate antigen presentation by lymphocytes, stimulate tumor-specific T cells, and activate systemic immunity, thereby enhancing the anti-tumor effect (75). It can be employed for the treatment of pancreatic cancers, PCLs, and NETs. In a meta-analysis, EUS-RFA exhibited technical and clinical success rates of 100 and 91.5%, respectively, for malignant pancreatic tumors and NETs, alongside an AE rate of 15% (76). In a study conducted by Jiang et al. (77) patients with advanced pancreatic cancer were treated by EUS-RFA. One month after the procedure, tumor size reduced and no significant early AEs. However, this study included a limited number of patients. A recent article focused on analyzing the safety of EUS-RFA, revealing an AE rate of 8%, with 1% identified as severe AEs. There was no reported mortality (78). It has been demonstrated that RFA is safe and feasible. A study compared the use of EUS-RFA in combination with systemic chemotherapy versus chemotherapy alone for the treatment of unresectable pancreatic cancer. The findings indicated that the combination therapy resulted in a higher tumor necrosis rate and reduced the need for pain relief. Although there was no statistically significant difference in the average tumor diameter between the two groups before and after treatment, the average tumor diameter increased after treatment in the chemotherapy alone group, suggesting that the combination therapy has an inhibitory effect on tumor growth (79). In terms of functional pancreatic NETs, a PSM study comparing EUS-RFA to surgery revealed a significantly lower rate of AEs in the EUS-RFA group compared to the surgical group (18.0% vs. 61.8%, *p* < 0.0001), while maintaining a similarly high clinical efficacy (95.5% vs. 100%) (80). Both EUS-EA and RFA can be utilized for ablating NETs and cystic lesions within the pancreas. Garg et al. (81) conducted a comparative analysis of the clinical and technical success rates, as well as AEs, associated with the two techniques for pancreatic NETs, revealing no substantial differences. However, EUS-RFA demonstrates a higher complete response (CR) rate for tumors (82). Conversely, EUS-EA exhibits a higher CR rate but has a greater frequency of AEs in cases of cystic lesions (73). Currently, there is limited evidence to determine which is better, and more research is needed to prove this point. Both methods are progressively emerging as complementary treatments for the multidisciplinary management of pancreatic tumors (83). The widespread application of EUS-RFA in the treatment of pancreatic tumors has been hindered by the occurrence of AEs, including acute pancreatitis, thermal injury to organs, as well as technical limitations.

In contrast to the thermal ablation of EUS-RFA, the utilization of hybrid bipolar cryoprobes has revealed the potential for combining RFA with low-temperature induction, resulting in anti-tumor effects. Petrone et al. (84) were the first to demonstrate the application of this technique, known as EUS-CTA, in a vitro study. Subsequent study has confirmed the safety and efficacy of it in patients with advanced pancreatic cancer (59).

EUS-PDT operates on the principle of employing EUS to precisely deliver photosensitizing drugs, which induces the generation of reactive oxygen species (ROS), consequently promoting cellular apoptosis and augmenting the permeability of tumor tissue (85). Choi et al. (60) in 2015, used EUS-PDT in patients with advanced pancreaticobiliary malignancy, whereby they achieved a median necrotic volume of 4 cm^3^. DeWitt et al. (64) conducted a prospective study involving 12 patients with advanced pancreatic cancer who received combined treatment of EUS-PDT with paclitaxel or gemcitabine. Out of the cohort, 6 patients had focal tumor necrosis, of whom one patient achieved CR as confirmed by postoperative pathology, no complications were reported. Although limited clinical data support its role as a palliative treatment method for advanced pancreatic cancer, current research indicates that EUS-PDT is a safe and feasible intervention for pancreatic cancer.

Di Matteo et al. (63) pioneered the use of EUS-LA to treat patients with unresectable pancreatic cancer. In their study, they performed EUS-LA on a cohort of 9 patients, utilizing a power range of 2–4 watts and delivering energy in the range of 800–1,200 Joules. The largest observed ablation area, measuring 6.4 cm^3^, was achieved at the power setting of 4 watts/1000 Joules, among the patients who underwent the procedure. Notably, no AEs occurred during the treatment, affirming the feasibility of this approach. Following this, Lim et al. (86) conducted a study using a 1,064 nm laser system that emitted a laser output of 5 watts in a porcine model of pancreatic cancer. They found that EUS-LA induced acute coagulation necrosis in the targeted pancreatic tissue. The size of the ablation zone showed a linear correlation with the total energy delivered. Initially, the effective ablation area demonstrated an increasing trend with the rise in total energy, eventually reaching saturation at 600 Joules. Nevertheless, additional experimental evidence is necessary to assess the presence of inflammatory reactions in the surrounding normal pancreatic tissue, as this finding would be pivotal in establishing the clinical safety and efficacy of EUS-LA.

## 5 Endoscopic ultrasound-guided fiducial implantation and endoscopic ultrasound-guided brachytherapy

Radiotherapy is a crucial therapeutic modality for pancreatic cancer, but its high-dose radiation may result in damage to nearby organs. To mitigate this potential harm, markers can be used for localization in pancreatic cancer radiotherapy. These markers, comprised of gold, platinum, or carbon, can be surgically or percutaneously inserted into the target lesion with the assistance of ultrasound, CT, or EUS. Subsequently, these markers can undergo low-dose gamma radiation, X-ray, or beta particle irradiation, resulting in localized tissue injury and tumor ablation (87). EUS is widely regarded as the preferred technique for marker implantation in pancreatic lesions due to its minimal invasiveness and ability to achieve the closest proximity to the pancreas (88–90). A meta-analysis demonstrated an overall success rate of 96.27%, with a particle migration rate of 4.33% and an AE rate of 4.85% for marker implantation guided by EUS (91). The conventional approach to marker implantation entails an initial lesion puncture using a biopsy needle, followed by subsequent marker implantation through the same needle. A study was conducted to compare the utilization of a preloaded 22-gauge (22G) needle with two markers to the conventional approach of placing a single marker using a 19G needle, assessing the technical success rate, clinical success rate, average procedure time, and occurrence of AEs. Similar success rates and AEs rates were observed between the two approaches, but the 22G needle enabled more markers, with 78% of patients receiving four markers, as opposed to only 23% with the 19G needle. These findings provide evidence of the feasibility and safety associated with the utilization of the preloaded 22G needle (92). However, further research is needed to establish the benefits of mortality reduction between two methods.

EUS-guided brachytherapy is the intratumoral administration of radioactive particles. Previously, the radioactive particle used was 125I, but later on, 32P started to be applied in advanced pancreatic cancer (93, 94). In a study involving 23 patients with advanced pancreatic cancer, 32P with gemcitabine exhibited favorable tolerability and feasibility (95). An ongoing study exploring the efficacy of combining 32P, gemcitabine, with or without paclitaxel in advanced pancreatic cancer has demonstrated promising therapeutic outcomes, involving a total of 9 patients, all of whom underwent successful implantation of radioactive particles without any severe AEs (96). Further data is needed, but the study has successfully demonstrated the feasibility of EUS-guided fiducial implantation and its potential for combination with other treatment modalities. Recently, a multicenter prospective study assessed the efficacy of combining 32P with 5-fluorouracil, leucovorin, irinotecan and oxaliplatin (FOLFIRINOX) or gemcitabine/nab-paclitaxel chemotherapy in patients with advanced unresectable pancreatic cancer. The study showed no significant AEs associated with 32P. Importantly, the tumor size, metabolic response, and CA19-9 levels significantly decreased, resulting in a local disease control rate of 90.5% after 16 weeks. Encouragingly, 23.8% of patients achieved a transition from unresectable to potentially operable after 32P treatment, with a MST of 15.5 months, surpassing that of other studies employing percutaneous implantation of 32P (94). However, the absence of a control group in this study necessitates further research to evaluate OS.

## 6 Endoscopic ultrasound-guided celiac plexus block and neurolysis (EUS-CPB/CPN)

The selection of analgesic therapy for chronic pancreatitis and pancreatic malignancy is a frequent dilemma in clinical practice. Despite the frequently inadequate results of drugs, EUS-CPB and CPN are effective therapeutic options for pain management in patients suffering from chronic pain. The transmission of pancreatic pain occurs through the celiac plexus, which conveys pain signals from the periphery to the central nervous system, leading to pain perception. CPB and CPN are performed using a linear echoendoscope through which a 19 or 22 G needle is passed and used for drug delivery. CPB administrates corticosteroids and long-acting local anesthetics via injection into the celiac plexus. Conversely, CPN typically involves the injection of substances, such as alcohol or phenol, that exert a more prolonged effect (97). For EUS-CPB in chronic pancreatitis, the American Gastroenterological Association (AGA) guideline does not recommend routine implementation. However, for patients with debilitating pain that remains unresponsive to other therapy, individual considerations may be warranted. Nevertheless, CPB should only be made after a thorough discussion of the uncertain outcomes and procedural risks associated with this intervention (98). Currently, CPN primarily includes bilateral approach, central approach, direct ganglion injection, and broad approach (99–101).

The guideline recommends the bilateral approach as the preferred method for CPN, but technical feasibility and operator comfort justify central injection as an acceptable option (strong recommendation, moderate-quality evidence) (102). Comparative studies have shown that both the bilateral and central approaches yield similar outcomes in terms of pain control. However, most research indicates a substantial reduction in opioid dosage with the bilateral approach (103–105). Some studies with conflicting opinions, indicating that the bilateral approach does not reduce the opioid dosage and the central approach exhibits a higher rate of pain relief compared to the bilateral approach (66% vs. 57%) (106, 107). In a prospective study comparing direct ganglion injection with the central approach, it was found that patients receiving direct ganglion injection had a shorter MST of 5.59 months compared to 10.46 months in the central approach, but no significant improvements were observed in pain relief rates, quality of life, or AEs (108). According to a meta-analysis, ganglion injection demonstrates superior pain control in the short term when compared to alternative methods in EUS-CP. However, it may lead to decrease in OS, and the long-term efficacy of pain control remains inconclusive (109). Combining ganglion injection with the central approach demonstrates superior rates of pain relief compared to the central approach alone (101). Furthermore, the broad approach exhibits great pain control compared to the bilateral approach (110), and when combined with ganglion injection, it demonstrates even better pain control (111). Koulouris et al. (100) conducted a systematic review and meta-analysis assessing the efficacy of the first three EUS-CPN techniques in pain control. At the 2-week follow-up, 68% of patients achieved pain control, while at the 4-week follow-up, this percentage decreased to 53%. Although there were no significant differences in success rates among the three methods, the central approach stood out as the only one without severe complications. It may indicate that central approach is the simplest way to operate. However, this review may have significant bias due to the inclusion of low-quality non-randomized studies and the absence of assessment regarding other treatments effects (e.g., opioid medications or chemotherapy).

The optimal timing for EUS-CPN remains unclear. If patients experience effective pain relief with opioid medications, CPN may not be necessary. On the contrary, early combination with CPN may be considered (112). The optimal timing should be based on a comprehensive assessment of the patient’s risks and benefits, expectations for pain control, stages of the disease, and individual clinical circumstances. The effectiveness of EUS-CPN in pain control varies from 50 to 94% in different studies, with a duration of pain relief ranging from 4 to 8 weeks (99). Recently, a tertiary hospital in India demonstrated EUS-CPN with a 100% technical success rate, suggesting that this procedure is easy and effective, rendering it suitable for broad implementation (113).

## 7 Discussion

EUS provides close proximity to the tissue, reducing interference from subcutaneous tissue, bones and gas. The utilization of high-resolution, real-time imaging facilitates the identification of minute lesions. As a consequence of these unparalleled advantages, EUS has emerged as a primary diagnostic approach for pancreatic lesions (2).

With the continuous development of endoscopic technology and equipment, EUS has transitioned from a diagnostic method to a minimally invasive intervention. In the management of PFCs drainage, plastic stents are progressively being substituted by barbell-shaped metal stents. Presently, a electrocautery-enhanced LAMS (Hot-Axios) has been employed in clinical practice (114). This stent allows stent deployment in a 1-step procedure guided by EUS. This system avoids utilization of additional accessories (such as needles, guidewires, or dilator devices), thereby reducing the overall procedural complexity and minimizing the risk of AEs (115, 116). The effectiveness and safety of it have been demonstrated in PFCs drainage, with technical and clinical success rates both reaching 96% (117). Recently introduced in China, this stent has been employed by Chinese researchers in 15 patients with pseudocysts and 15 patients with walled-off necrosis, resulting in a technical success rate of 100% and a clinical success rate of 93.3%. After one month, the stent was successfully removed in 73.3% of patients. Patients with a history of pancreatitis lasting more than 6 months are more likely to achieve complete resolution of the lesion within one month of undergoing AXIOS treatment (118). Another electrocautery-enhanced LAMS (Hot-Spaxus), has been utilized for draining PFCs. It demonstrates similar clinical and technical success rates as Hot-Axios, but its advantages stem from its prolonged length and reduced risk of bleeding (119). Larghi et al. (120) deployed Hot-Spaxus for PFCs drainage, achieving technical success in all cases without any AEs. However, there have been few comparative studies on this topic, and there is a lack of clear evidence associating other types of LAMS with an increased risk of bleeding. With the advent of new stents, drainage is no longer limited to lesions within 10 mm of the gastric or duodenal wall. LAMS can effectively drain even lesions measuring 10-14 mm, providing a clinical and technical success rate of 97% (121). The debate over the potential risk reduction of AEs, such as bleeding, with the coaxial placement of metal and plastic stents remains unsettled, warranting further prospective studies to provide substantiating evidence (34, 37).

EUS-guided ablation techniques have primarily been utilized in the management of PCLs and neuroendocrine tumors, employing a diverse range of procedural approaches, amidst the progress of precision medicine and translational research. In specific cases of non-functional small pancreatic neuroendocrine tumors, EUS-EA may be preferred over SR (66). Nevertheless, EUS-EA cannot obtain histopathological confirmation of the disease type. Continued advancements are required to optimize needle size, ethanol injection concentration, ablation techniques, and treatment effect, aiming to enhance efficiency while minimizing AEs. Moreover, its combination with chemotherapeutic agents in conjunction with laser ablation or photodynamic therapy has been explored for the management of advanced pancreatic malignancy, despite limited evidence supporting its feasibility (63, 64). The differences in treatment CR and AEs between EUS-EA and EUS-RFA for solid pancreatic tumors and cystic lesions may be associated with the limitedly existing research on cystic lesions, thus necessitating additional literature to provide further substantiation. Lim et al. (86) recently reported that EUS-LA induces acute coagulative necrosis in the targeted tissues of porcine pancreatic cancer. More study is required to verify if the normal pancreatic tissue exhibits inflammatory reactions, crucial in determining the clinical safety of the procedure. Nevertheless, the efficacy of this procedure for pancreatic lesions has already been established. In the management of advanced pancreatic tumors, precise therapies such as EUS-FNI, EUS- guided fiducial implantation, and EUS-guided brachytherapy are progressively being integrated into clinical practice, aiming to reduce radiation and enhance treatment precision. Recent studies have proved the effectiveness of EUS-FNI in alleviating adverse events and exhibiting promising therapeutic outcomes for pancreatic cancer treatment (46). In addition, EUS-guided brachytherapy has demonstrated a disease control rate of over 90% and provides a surgical option for unresectable pancreatic cancer (94). Recent studies have shown the efficacy of a combination therapy in a mouse model of pancreatic cancer, where thermally responsive elastin-like polypeptide (ELP) conjugated with iodine-131 radionuclides (131I-ELP) were delivered through nanoparticle albumin-bound paclitaxel. This intervention induces alterations in the expression of intercellular collagen and adhesion proteins within the tumor microenvironment, leading to complete regression of pancreatic cancer (122). However, pancreatic cancer is a systemic disease, and it is highly improbable that the aforementioned treatments will completely replace current therapies for pancreatic cancer. Nevertheless, considering the safety and feasibility of the mentioned methods, as well as the expansion of translational medicine, they might become viable adjunctive approaches in the future. Further development of these approaches could involve the injection of alternative drugs, combination of brachytherapy and chemotherapy, among other possibilities. They are hopeful for precision treatment of pancreatic cancer patients. It is hoped that future clinical research can increase the proportion of pancreatic cancer patients who can undergo SR or achieve CR.

Effective pain management is crucial for patients with advanced pancreatic tumors and chronic pancreatitis, as it can improve the quality of life for those with cancer and alleviate suffering in individuals with chronic diseases. An ongoing debate persists regarding the optimal approach to achieve pain relief in EUS-CPB/CPN. Current studies suggest that central with ganglion injection could potentially be the most appropriate method (101). According to the AGA guideline, EUS-CPB and CPN are not routinely recommended for pain management in patients with chronic pancreatitis and advanced pancreatic cancer. However, they can serve as adjunctive methods for patients who prefer not to undergo surgery or wish to reduce their use of opioid medications (98). Some researches indicate that the utilization of opioid medications can prolong the MST in pancreatic cancer patients from 4 months to 6 months (123). Conversely, patients who receive CPN exhibit a shorter survival time (4 months) in contrast to those who use opioid medications (7 months) (124). Consequently, the EUS-CPN remains a topic of debate.

In general, the development of EUS has made remarkable strides in the diagnosis and treatment of pancreatic diseases. In recent decades, a variety of EUS-related techniques have undergone continuous updates and evolution, with the incorporation of multiple therapies propelling technological advancements. This dynamic interplay with diverse therapeutic approaches has reinvigorated EUS and shaped its trajectory in the management of pancreatic diseases. The aforementioned treatment approaches have been advancing toward simplicity and minimally invasive procedures, aiming to enhance patients’ quality of life and survival rate, thereby yielding substantial accomplishments. Moving forward, EUS will require endoscopists to attain higher levels of proficiency. Despite the current limited long-term evidence or prognostic data on certain EUS-related techniques, their application in pancreatic diseases is anticipated to gain broader acceptance as they evolve, fostering deeper integration and dissemination of various adjunctive technologies, ultimately resulting in increased patient benefits.

## Author contributions

RX: Writing – original draft. KZ: Writing – review & editing. NG: Writing – review & editing. SS: Supervision, Writing – review & editing.

## Figures and Tables

**Table:** 

EUS	Endoscopic ultrasound
EUS-FNI	EUS-guided fine-needle injection
EUS-CPB	EUS-guided celiac plexus block
EUS-CPN	EUS-guided celiac plexus neurolysis
PFCs	Pancreatic fluid collections
WON	Walled-off necrosis
AEs	Adverse events
DPPS	Double pigtail plastic stents
SEMS	Self-expanding metal stent
LAMS	Lumen-apposing metal stent
ASGE	American Society for Gastrointestinal Endoscopy
OS	Overall survival
MST	Median survival time
PFS	Progression-free survival
CHST-15	Carbohydrate sulfotransferase-15
EUS-EA	EUS-guided ethanol ablation
EUS-RFA	EUS-guided radiofrequency ablation
EUS-CTA	EUS-guided cryothermal ablation
EUS-PDT	EUS-guided photodynamic therapy
EUS-LA	EUS-guided laser ablation
NETs	Neuroendocrine tumors
DSS	Disease-specific survival
CR	Complete response
ROS	Reactive oxygen species
22G	22-gauge
FOLFIRINOX	5-fluorouracil, leucovorin, irinotecan and oxaliplatin
AGA	American Gastroenterological Association
ELP	Elastin-like polypeptide
EGD	Esophagogastroduodenoscopy
RCTs	Randomized controlled trials
PSM	Propensity score-matching
BD-IPMNs	Branch-duct intraductal papillary mucinous neoplasms
SR	Surgical resection
PCLs	Pancreatic cystic lesions
NA	Not available
PR	Partial response
LAPC	Locally advanced pancreatic cancer
PC	Pancreatic cancer
PCN	Pancreatic cystic neoplasm
ESGE	European Society of Gastrointestinal Endoscopy
